# The Role of Interferons in the Pathogenesis of Sjögren’s Syndrome and Future Therapeutic Perspectives

**DOI:** 10.3390/biom11020251

**Published:** 2021-02-09

**Authors:** Nicoletta Del Papa, Antonina Minniti, Maurizio Lorini, Vincenzo Carbonelli, Wanda Maglione, Francesca Pignataro, Nicola Montano, Roberto Caporali, Claudio Vitali

**Affiliations:** 1 Department of Rheumatology, ASST G. Pini-CTO, 20122 Milano, Italy; antonina.minniti@asst-pini-cto.it (A.M.); wanda.maglione@asst-pini-cto.it (W.M.); francesca.pignataro@asst-pini-cto.it (F.P.); roberto.caporali@unimi.it (R.C.); 2 Department of Clinical Sciences and Community Health, Ca’ Granda IRCCS Foundation, Ospedale Maggiore Policlinico, Università degli Studi di Milano, 20122 Milano, Italy; maurizio.lorini@unimi.it (M.L.); vincenzo.carbonelli@unimi.it (V.C.); nicola.montano@unimi.it (N.M.); 3 Research Center for Adult and Pediatric Rheumatic Diseases, Department of Clinical Sciences and Community Health, Università degli Studi di Milano, 20122 Milano, Italy; 4 Mater Domini Humanitas Hospital, Rheumatology Outpatient Clinics, 21053 Castellanza, Italy; claudio.vitali@yahoo.it

**Keywords:** type I interferon, type II interferon, Sjögren’s syndrome, target therapies

## Abstract

There is a great deal of evidence pointing to interferons (IFNs) as being key cytokines in the pathogenesis of different systemic autoimmune diseases, including primary Sjögren’s syndrome (pSS). In this disease, a large number of studies have shown that an overexpression of type I IFN, the ‘so-called’ type I IFN signature, is present in peripheral blood mononuclear cells, and that this finding is associated with the development of systemic extra-glandular manifestations, and a substantial production of autoantibodies and inflammatory cytokines. In contrast, the absence or a milder expression of type I IFN signature and low level of inflammatory cytokines characterizes patients with a different clinical phenotype, where the disease is limited to glandular involvement and often marked by the presence of widespread pain and depression. The role of type II (IFNγ) in this subset of pSS patients, together with the potentially related activation of completely different immunological and metabolic pathways, are emerging issues. Expression of both types of IFNs has also been shown in target tissues, namely in minor salivary glands where a predominance of type II IFN signature appeared to have a certain association with the development of lymphoma. In view of the role played by IFN overexpression in the development and progression of pSS, inhibition or modulation of IFN signaling has been regarded as a potential target for the therapeutic approach. A number of therapeutic compounds with variable mechanisms of action have been tested or are under consideration for the treatment of patients with pSS.

## 1. Introduction

Primary Sjögren’s syndrome (pSS) is a systemic autoimmune disease predominantly affecting post-menopausal women. The pathologic hallmark of pSS is the focal lymphocytic infiltrations in salivary and lachrymal glands. Chronic inflammatory changes in the glands induce a decline in their secretory function with the consequent symptom of dry eyes (xerophthalmia), associated with the features of keratoconjunctivitis sicca, and dry mouth (xerostomia). In around 50% of the patients, the clinical symptoms related to glandular involvement (GI) are accompanied by extra-glandular manifestations (EGMs) that mainly involve joints, kidney, lung, peripheral nervous systems, and small vessels [1]. Severe fatigue and widespread pain (WP) are other characteristic features of the disorder [2], often associated with a depressive state.

The pathogenesis of pSS is largely unknown. Similar to other autoimmune diseases, pSS is a complex disorder where the development of the pathological process requires the intervention of different factors. Among these, some environmental factors, for instance an incident viral infection, may trigger the disorder [3,4]. Although no specific infectious agent has been identified as a certain inducer of the development of pSS, disease manifestations that may simulate those of pSS have been observed in a variety of infections. In past years, different viruses, such as hepatitis C virus (HCV), human immunodeficiency virus (HIV), Epstein–Barr virus (EBV), cytomegalovirus (CMV), coxsackievirus, and human T-lymphotropic virus-1 (HTLV-1), have been evoked as possible triggers of pSS, since these viruses may cause persistent infection of the salivary glands and lead to organ destruction, thus causing sicca syndrome of the mouth [3,4]. However, some pathological aspects of the sialoadenitis caused by these viral infections are consistently different [5].

Particular interest has been raised by HTLV—a retrovirus which has been reported to be involved in the pathogenesis of primary SS in endemic areas of Japan. HTLV-I preferentially infects T cells, especially CD4+ T cells, but it can also infect salivary epithelial cells, although with a low capacity of forming ectopic germinal centers and destroying glandular tissue [6].

In past years, evidence has accumulated that supports a significant relationship between the ‘so-called’ Human Endogenous Retroviruses (HERVs) and the development of different autoimmune diseases. For millions of years, HERVs have been integrated in human genomes. The HERV sequences are usually silent or perform some physiological roles but may also become active and influence the development of a variety of chronic diseases, including autoimmune diseases [7]. The presence of the HERV sequences has also been reported in significantly higher number of patients with pSS, in comparison with healthy controls (HCs) [8].

It is commonly believed that whatever the infectious agent which might trigger the development of pSS, it can more easily do it in the presence of a susceptible genetic background. Genome-wide association studies (GWAS) have identified alleles belonging to class II major histocompatibility complex (MHC), namely those of the Human Leukocyte Antigen HLA-DR and HLA-DQ isotypes, which are closely associated with pSS. In addition to these human leukocyte alleles, several non-MHC genes have also shown a significant association with the disease. Some of these susceptibility genes, such as Interferon regulatory factor 5 (IRF5), signal transducer and activator of transcription 4 (STAT4), and Interleukin 12A (IL12A), take part in the transmission of interferon (IFN) signaling [9,10]. Furthermore, gene polymorphism of some genes belonging to the IFN pathway has also been reported in pSS [11,12,13]. In the last decades, an upregulation of a number of IFN-stimulated genes (ISGs), the so-called ‘IFN signature’, has been described in peripheral blood and target tissues of a large number of patients with pSS [14]. Thus, it has been postulated that these upregulated genes may play important roles in the pathogenesis of pSS by activating some crucial biological processes of the disease [15]. However, both IFN pathway activation and polymorphism of IFN signaling-related genes are not specific for pSS but are shared by other systemic autoimmune disorders [16].

## 2. Biological Properties of IFNs

Three different types of IFNs have been described: type I, type II, and type III, of which type I IFNs make up the largest group. Five classes of type I IFN have been identified, i.e., IFN-α, IFN-β, IFN-ε, IFN-κ, and IFN-ω [17]. IFN-α and IFN-β are the most extensively studied type I IFNs subtypes in rheumatic diseases, including pSS. Consequently, most of the biological mechanisms which have been ascribed to type I IFN signaling have been demonstrated in studies limited to IFN-α and IFN-β [18].

IFN-α and IFN-β, and probably most of the other type I IFN subtypes, interact with IFNα/β receptor (IFNAR), consisting of two subunits, IFNAR1 and IFNAR2. As a result of this binding, auto-phosphorylation of Janus protein kinases 1 (JAK1) and tyrosin kinase 2 (Tyk2) occurs, and, as a second step, STAT1 and 2 are then activated, leading to the formation of a STAT2/STAT1 heterodimer. The subsequent binding to interferon-regulatory factors (IRFs), and namely IRF3 and IRF7, induces the formation of IFN-stimulated gene factor 3 (ISGF3) [14,17]. The latter element translocates to the nucleus and promotes the activation and transcription of IFN-stimulated genes (ISGs). The proteins encoded by ISGs are characterized by a wide variety of antiviral and anti-neoplastic properties [17,19], and by several immunomodulatory effects, such as the induction of B cell activating factor (BAFF), immunoglobulin switching, increased antigen presentation, T-cell-mediated, and natural killer cell (NK) cytotoxicity [20,21]. To avoid an excess of immune stimulation, a regulatory control of type I IFN signaling is warranted by post-transcriptional modifications of the type I IFN pathway and by downregulation of type I IFN expression via the epigenetic modifications, including DNA methylation, histone modification, and non-coding RNA interactions [22]

Any cell is considered virtually able to produce type I IFNs [23], following stimulation by exogenous or endogenous nucleic acids. However, it has been shown that plasmacytoid dendritic cells (pDCs) are the main IFNα-producing cells. Many studies have reported that these cells possess an increased expression of Toll-like receptor (TLR)-7 and TLR-9, which makes these cells particularly sensitive to stimulation from a variety of endogenous and exogenous elements, such as single stranded-RNA (ss-RNA)/endogenous RNA and unmethylated DNA. These nucleic acid products selectively bind these receptors and induce a robust type I IFN stimulation [24,25].

Type II IFN (IFN-γ), and the more recently described in humans type III IFNs, bind different receptors and have a low degree of homology with type I IFN. However, all these IFN types interact with the different IFN binding receptors and, via the JAK-STAT pathway, induce largely overlapping genes [26,27,28].

IFN-γ is mainly produced by T lymphocytes and natural killer (NK) cells, although other immune cells are also able to produce this cytokine [29]. It is involved in several aspects of immunity, including activation of macrophages and T helper (Th) lymphocytes, antigen presentation, cell proliferation and apoptosis, induction of antiviral state, and leukocyte trafficking [27,29,30]. IFN-γ drives the polarization of macrophages towards an M1 profile. The hallmark of this subtype of macrophages is the production of large amounts of pro-inflammatory cytokines [31]. IFN-γ has the same effect on the polarization of Th cell populations, since, together with IL-12, it induces the differentiation of naïve T-lymphocytes into Th1 cells [32], which are the greatest producer of pro-inflammatory cytokines.

IFN-γ, after binding with its specific receptors (IFNGR1 and 2), triggers the JAK/STAT signaling pathway [27,29]. JAK1 and JAK2, which are constitutively associated with IFNGR1 and IFNGR2 respectively, are then phosphorylated with the consequent activation of STAT1 as a transducer signal. STAT1 phosphorylation induces the activation of the promoter region of IFN-γ-induced genes [29,30].

Different cytokines modulate the production of IFN-γ. After an infection, antigen-presenting cells (APCs) secrete IL-12, IL-18 and IL-1β, which enhance the IFN-γ synthesis [29,33]. In contrast IL-4, IL-10, transforming growth factor-beta (TGF-β) and glucocorticoids downregulate its production [29]. Finally, it has been demonstrated that IFN-γ induces vascular cell adhesion molecule expression. This facilitates the homing of additional inflammatory cells to the involved tissues, and then the perpetuation of the local inflammatory process [34]

Type III IFN is composed of three distinct molecules, IFN-λ1/IL29, IFN-λ2/IL-28A, and IFN-λ3/IL-28B, which all bind a common receptor (IFN-λR1/IL-28Ra). They are expressed mainly by mucosal epithelia in barrier tissues and by several types of immune cells. Type III IFN molecules also possess anti-viral activity and protect epithelial surfaces of the gut, lung, urogenital, gastrointestinal tract, and liver [35]. Furthermore, other data indicate that these molecules play some role in the regulation of both innate and adaptive immune responses in a manner similar to that of type I IFNs.

**Figure 1:**
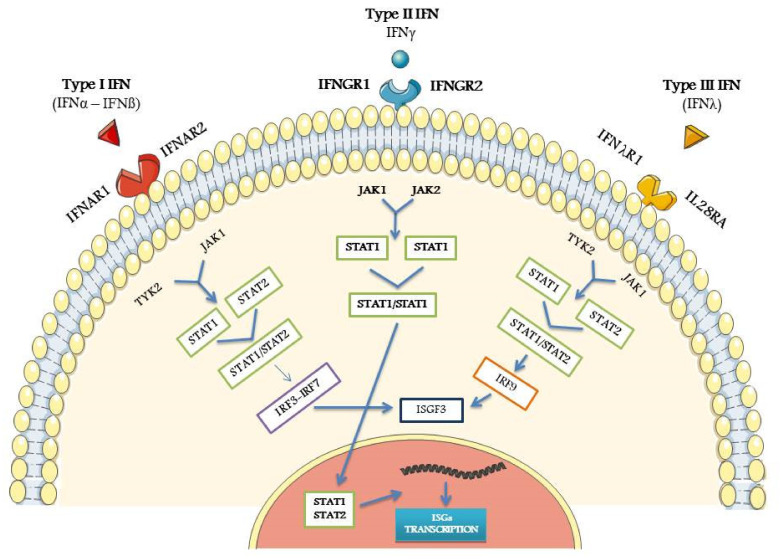
Schematic view of the main steps of type I, type II, and type III Interferon-IFN pathways, from the specific receptor link to IFN-stimulated genes (ISGs) activation. For all the abbreviations, see text. Credit: modified from Smart Servier Medical Art (https://smart.servier.com (accessed on 18 January 2021)).

Figure 1 shows a simplified schematic view of the IFNs signaling pathways.

## 3. Detection of IFN-Related Activity

Measurement of IFNs in peripheral blood is a very difficult challenge. Old commercial enzyme-linked immunoassay (ELISA) tests gave uncertain and contradictory results in assessing the presence and level of IFNα molecules in the serum of patients with autoimmune diseases, probably because of the simultaneous presence of different IFN subtypes [36]. A more advanced ELISA array based on a novel digital technology has shown promising results, but its use is still limited to other disorders and has not been applied in pSS so far [37].

Currently, transcriptional analysis of IFN-induced genes is the most used method to demonstrate an upregulation of IFNs in patients with different autoimmune diseases [38,39,40]. A significant amount of data obtained using these methods has also shown that an upregulation of type I IFN-induced genes is present in peripheral blood mononuclear cells (PBMCs), isolated monocytes, and target tissues of pSS [41,42,43]. This finding has been defined as ‘type I interferon signature’. Furthermore, a restricted number of genes predominantly or exclusively induced by type I IFN was selected and used to assess the presence of type I IFN signature, and then to calculate the ‘so-called’ type I interferon score, which is derived from the mean level of overexpression of these selected genes in comparison to that observed in HCs [42,44].

Although type I and II IFNs induce the expression of largely overlapping groups of genes, precise probes for IFN-γ activity have also been identified in a variety of autoimmune disorders [45]. The ability to define differences between the type I and type II IFN signatures may help to better identify the biological processes which mainly drive the different phases of the disease or predominate in the different subsets of patients.

## 4. Role of IFNs Signature in the Pathogenesis of pSS in Humans

Microarrays and real-time quantitative polymerase chain reaction (RT-qPCR) studies demonstrated that an upregulation of ISGs was evident in minor salivary gland (MSG) biopsies and ocular epithelial cells of patients with pSS, compared to HCs [40,43]. However, when specific probes for either type I or type II IFN transcripts (IFIT-3 and GBP-2, respectively) were used, it was clear that expression of type I and type II IFN at the MSG level was not similar in all the patients and may vary in different phases of the disease [45]. Specific type I-related transcript (IFIT-3) was mainly localized in salivary duct epithelial cells, whilst a type II-related transcript (GBP-2) was found in both lymphoid aggregates and duct epithelial cells localized in the site of inflammatory cell infiltration [46]. By using immunoblot analysis on gland lysates and immunohistochemical techniques on gland sections, it was also possible to distinguish three IFN patterns in salivary gland tissue, i.e., predominantly type I or type II, or a mixed type I/II IFN expression. However, there were no differences in the clinical features between patients subdivided according the three IFN patterns observed in their MSGs, but a higher focus score was reported in type II predominant patients [46]. The fact that in some patients a type II IFN signature is predominant in the MSGs was confirmed in a study showing increased IFNγ but low IFNα transcripts in this tissue. This is particularly evident in salivary glands from pSS patients with lymphoma, compared to those obtained from pSS patients without lymphoma and non-SS sicca controls [14]. Consequently, the IFNγ/IFNα ratio has been proposed as a potential marker to predict lymphoma development among SS patients [47].

Apart from IFN signature in MSG tissue, an increased expression of type I IFN inducible genes or proteins has also been revealed in PBMCs [41,48], pDC [49], and B-cells [50]. Type I IFN signature was also found in isolated CD14 monocyte and this finding was associated with higher disease activity level, more pronounced production of autoantibodies, and enhanced B-Cell-Activating Factor (BAFF) gene expression [42].

In another study where a modular transcriptional repertoire methodology previously used in lupus patients was applied [51], three distinct IFN patterns were observed in peripheral blood from pSS patients, a type I IFN predominant, a type I and II IFN mixed pattern, as well as an inactive one [52]. Total European League Against Rheumatism (EULAR) SS Disease Activity Index (ESSDAI) [53] or Clinical ESSDAI (ClinESSDAI) [54] did not differ between the three groups, with the exception of the ESSDAI biological domain score that was higher in the patients characterized by type I and mixed IFN signature. There were no differences in the EULAR SS patient-reported index (ESSPRI) [55] regarding fatigue or dryness between groups, but pain scores were lower in the IFN-active groups [52].

A different pattern of IFN signature in patients with a high pain score has been confirmed in a study by our group [56], in which RT-qPCR analysis, using specific probes, was used to investigate the type I and type II IFN-related gene expression in PBMCs. Four IFN type I and five IFN type II predominantly induced genes were selected to perform this study in patients with pSS and in healthy controls (HCs). Out of the enrolled patients with pSS, 11 were characterized by the presence of a number of EGMs and significantly higher ESSDAI score, and 10 patients by the absence of EGMs and the presence of WP, associated with a significantly higher ESSPRI pain score. The ESSPRI fatigue score did not differ between the two groups. The fold change values of gene expression, in comparison with HCs, were double-normalized using the 2−ΔΔCT method [57]. As Table 1 shows, fold change values of IFN type I-induced genes appeared much higher in patients with EGMs, and also moderately overexpressed in patients without EGMs, but with WP. Only indoleamine 2,3-dioxygenase 1 (IDO1) among the type II IFN-related genes is slightly increased in patients with WP. These data indicate that IFN type I- and, to a lesser degree, type II-induced genes are upregulated in the PBMCs of patients with pSS, but this phenomenon is almost completely restricted to patients with systemic EGMs. Finally, it is noticeable that the IDO1 gene in this population is strongly upregulated in patients with EGMs and, to a lesser degree, overexpressed in patients with WP [56].

IDO is an enzyme encoded by two homologous genes, IDO1 and IDO2. IDO1 is predominantly upregulated by IFNγ but also by other cytokines such as IFNα, IL1, and TNFα. The IDO1 enzyme shifts the tryptophan (Tp) from the production of serotonin and induces the metabolic conversion of this amino acid to kynurenine (Kyn), which is further catabolized to other metabolites such as kynurenic acid, 3-hydroxy-anthranilic acid, and quinolinic acid [58]. Both Tp depletion and catabolites of the Kyn pathway suppress T cell activation and activate T regulatory cells (Tregs) [59]. The IDO1 gene and protein levels have been shown to be elevated in serum, pDCs, monocytes, and T cells of pSS patients [60,61]. In view of the role of the Tregs in modulation of the immune response, it has been suggested that the activation of the IDO1 pathway works as a counter-regulatory mechanism aimed at restoring the immune balance and inducing tolerance in pSS [61,62].

It has been demonstrated that IDO1 is also overexpressed in a vast majority of cancer cells. The induction of Treg-related immune tolerance is responsible for the suppression of the function of effector T and NK cells, and then may facilitate the progression of solid tumors [63]. So, targeting IDO1 is presently believed to be an interesting option for the immune therapy in oncology [63]. In contrast to what happens in cancer, in pSS, there is a strong activation of T effector cells, namely Th1 and Th17, not sufficiently modulated by Treg cells. Although studies on the Treg cell presence in pSS have often given contradictory results, probably because of the use of completely different assessment methodologies, some of these investigations have shown a reduction of Tregs in peripheral blood and salivary glands of patients with pSS [64,65], associated with an increased severity of the clinical features [64].

On the other hand, the activation of the catabolic pathway induced by IDO1 interferes with serotonergic and glutamatergic neurotransmission in specific areas of the central nervous system (CNS) [66]. As a consequence of these metabolic effects, it has been postulated that the activation of IDO1 may be responsible for some manifestations, like hyperalgesia, WP, and depression, which are frequently encountered in patients with pSS [2,66]. This is supported by the evidence that IDO1 pathway activation, measured by the increased Kyn/Tp ratio in the peripheral blood, has also been observed in other conditions characterized by chronic pain, including fibromyalgia [66,67]. Finally, the increased production of Kyn and Kyn metabolites has been found to be associated with a number of neurological and psychiatric disorders [66].

In contrast with these data supporting the relationship between the IFN pathway and WP/depression features, fatigue—which is present in around 70% of patients with pSS—did not appear to have relationship with the IFN signature, the related cytokine production, or IDO1 activation [68,69,70]. Conversely, decreased levels of some proinflammatory cytokines have been demonstrated in fatigued patients with pSS [68,69]. A recent study, based on an advanced proteomic analysis, showed that only the serum concentration of the inflammatory mediator IL36a, a cytokine belonging to the IL1 family, was increased in fatigued patients. The other upregulated proteins in this subgroup of patients did not have apparently any role in the inflammatory process, but rather may act in neurotransmission processes [71].

Recent data indicate that type III IFN—namely IFNλ—may have a role in the pathogenesis of pSS. Increased IFN-λ2/IL-28A epithelial expression in MSGs and IFN-λ1/IL-29 levels in the periphery were detected in patients with pSS in comparison to sicca controls [72]. Moreover, in an in vitro study on human salivary gland ductal cell line, the addition of IFNλ1/IL-29 to IFNα induced a further increased expression BAFF- and CXCL10-related genes, and the prolongation of STAT phosphorylation. These results suggest that IFNα and IFNλ may have a synergistic effect in the pathogenesis of SS [72].

Although certainly not conclusive and sometimes contradictory in different reports, the large amount of data describing the complex role of IFNs, ISGs, and the related inflammatory and metabolic processes have undoubtedly contributed to expand the present knowledge on the pathogenesis of pSS. From the clinical point of view, at least two subgroups of patients can be distinguished in the clinical spectrum of this disease. At one pole, there are patients characterized by a predominant B cell activation with extensive autoantibody production, presence of a variety of EGMs, and Th1/Th17 polarization with the release of a large quantity of cytokines. It has been ascertained that this subgroup is predominantly marked by the type I IFN signature [73]. At the opposite pole, there are patients characterized by low prevalence of EGMs, absence or lower level of B activation, and consequently of autoantibodies, and a clinical picture limited to GI and often the presence of chronic pain and depression. One can suppose that type I IFN signature may be milder or absent in this subset and, conversely, type II IFN signature may be predominant and could induce a modulation of immune response via the Treg cell action, and activate other metabolic processes [66,67]. These two patterns of IFN expression are probably more clearly detectable when the analysis is limited to the peripheral blood. In salivary glands, the balance between type I and II IFN signature appear to be completely different [43]. In this target tissue, an overexpression of type II IFN signature has been reported to be present and associated with the development of lymphoma [47]. This different pattern is probably induced by the active participation in the local inflammatory process of other non-inflammatory cells, such as glandular epithelial cells [23]. The continuously increasing evidence of the differences of IFN expression in different subsets of patients with pSS supports the belief that any clinical phenotype may underlie a different pattern of immune and metabolic processes. A recent study on gene expression in patients subdivided according to their different phenotypes confirmed this assumption [74].

## 5. IFN Signature, from Human Studies to Animal Models

The evidence that the activation of different biological pathways may characterize the different clinical and pathological phenotypes of pSS is also confirmed in animal models. Many mouse models developing SS-like disease have been studied [75]. However, the role of IFN signature in these models has been investigated in a limited part of them [76]. Apart from the spontaneous development of diabetes, female non-obese diabetic (NOD) mice present with a typical SS-like syndrome characterized by decreased salivary flow, lymphocytic infiltrates in the glands, and production of specific autoantibodies [77]. A modified strain of NOD mouse (C57BL/6.NODAec1Aec2) also develops SS-like disease, but not diabetes. It has been shown that type I IFN-induced genes are expressed in salivary glands of NOD mice and NOD-derived strains. In contrast, there is no evidence for systemic IFN type I activation in these animal models [76]. The crucial role of type I IFN in the development of salivary gland involvement has been confirmed in another mouse model where IFNAR was deleted (B6.IFNR-/-) [78]. In this strain, in contrast with the wild type, the stimulation with poly(I:C), an analogue of double stranded-RNA (dsRNA), did not cause salivary gland hypofunction.

Autoimmune-prone mice have been used to mimic systemic IFN activation by external stimulation with different substances. In this respect, New Zealand Black/White (NZM) F1 mouse strain is the most studied model since it can develop an SS-like disease together with a lupus-like syndrome. In this model, the stimulation of TLR3 with poly(I:C) induced an IFN systemic activation [79].

Furthermore, it has also been reported that, in NOD mice, type II IFN (IFNγ) participates in the early phase of SS-like disease in glandular tissues [80]. These data have been confirmed in another mouse model (Ro60-immunized Balb/c) in which increased levels of IFNγ correlated with decreased salivary flow [81].

The crucial role of TLR stimulation in inducing IFN pathway activation has been largely demonstrated in other models [82]. Namely, TLR7 stimulation seems to be the key point, as shown in a particular mouse model (BXSB/MpJ-Yaa), where a gene modification introducing a TLR7 duplication induced the development of an autoimmune dacryoadenitis as a component of a SS-like disease, together with an exacerbation of lupus-like disease [83].

Thus, in addition to human surveys, also studies on animal models mimicking the different phenotypes of pSS may contribute to better understand the different underlying biological mechanisms and then identify more tailored therapeutic approaches.

## 6. Perspectives of IFN-Modulating Therapies in pSS

In view of the large amount of data demonstrating that IFN pathway activation plays an important role in the development and progression of the pathological process in pSS, IFNs, and namely type I IFN, have been identified as a potential target for the treatment of this disorder. Considering the present knowledge on the biological mechanisms that can trigger the IFN metabolic cascade and the activation of IFN-related ISGs, different specific targets have been identified as eligible for a therapeutic intervention able to modulate IFN overexpression in pSS.

Table 2 summarizes the different therapies targeting IFN pathways that have been investigated so far.

### 6.1. Therapies Preventing the Activation of TLRs

Hydroxychloroquine (HCQ) is an old drug widely in use in rheumatoid arthritis and systemic lupus erythematosus (SLE). In pDCs from lupus patients, HCQ was able to decrease type I IFN production, probably preventing the activation of TLR7 and TLR9 receptors [91]. However, a randomized double-blind controlled trial performed in patients with pSS (JOQUER, clinicaltrials.gov Identifier: NCT00632866) failed to improve the disease symptoms [92], although it was able to inhibit type I IFN inducible gene expression in treated individuals.

Another way to impair the activation of pDS-specific TLRs, and then to reduce IFN production, is to hinder the link to these receptors of stimulating materials, such as nucleic acids. RSLV-132, a Fc portion of a IgG1 fused with RNAse, the enzyme capable of degrading circulating RNA, was tested in lupus patients with interesting results [93]. A phase II trial carried out in patients with pSS demonstrated that this molecular construct was effective in reducing fatigue and expression of selected IFN-inducible genes [84].

Since the activation of immunoglobulin-like transcript 7 (ILT7), a receptor molecule expressed on the surface of pDCs [94], leads to downregulation of TLR7/TLR9-mediated IFN production, by inducing a preferential differentiation of pDCs to an APC phenotype [94,95], stimulation of ILT7 has been regarded as a potential therapeutic option in patients with autoimmune diseases characterized by type I IFN signature. An antibody stimulating ILT7 (MEDI7734) has been tested in patients with pSS. Although the study was completed, no results have yet been reported (NCT02780674).

### 6.2. Therapies Modulating IFN Pathway Activation in pDCs

Ligation of blood DC antigen 2 (BDCA-2), a pDC-specific antigen, strongly suppresses induction of type I IFN production from these cells [96]. BIIB059 is a humanized IgG1 monoclonal antibody (mAb) that specifically recognizes BDCA2 and induces its rapid internalization from the cell surface of pDCs with the subsequent inhibition of type I IFN-I production. In a phase I study (NCT02106897), BIIB059 was assessed in a cohort of patients with SLE and active skin disease. Its administration decreased expression of IFN response genes and reduced infiltrates in skin lesions [85]. At the moment, this kind of therapeutic approach has not yet been tested in patients with pSS.

Since TRAF family member associated NFκB activator (TANK)-binding kinase (TBK1) is an important signal in PBMCs, and namely in pDCs, able to activate IRF3 and IRF7, leading to the subsequent induction of ISGs, blocking this kinase was identified as a potential mechanism to indirectly inhibit the IFN pathway. BX795, a potent inhibitor of TBK1, has shown to be capable of reducing the expression of ISGs in PBMCs from IFN-positive patients with different systemic autoimmune diseases, including pSS. Its potential efficacy has not been tested in clinical trials so far [86].

### 6.3. Therapy against IFNα and Related Receptors

The use of monoclonal antibodies (mAbs) against IFNα (rontalizumab, sifalimumab) has been proven in phase II placebo-controlled trials in patients with SLE with moderate results. In the rontalizumab trial, treatment was associated with reduction of disease activity (DA) and number of flares, and decreased steroid use only in patients with low disease activity scores [87]. In the sifalimumub trial, the primary end point (global DA decrease) was reached in a significantly higher percentage of treated patients with respect to the placebo group [88].

Targeting IFN receptor (IFNAR) could be another option to interfere with the biological effects of type I IFN. AMG811, a monoclonal antibody against IFNγ, has been tested in patients with SLE in a phase Ib, randomized, multiple-dose escalation study (NCT00818948). This target therapy demonstrated favorable pharmacokinetics and acceptable safety profile but no evidence of clinical impact, although IFN-γ-associated biomarkers showed a significant decrease [89]. 

Anifrolumab, a mAb targeting IFNAR, has been tested in three randomized controlled trials in SLE with a high IFN signature. This therapeutic approach demonstrated to be affective at least in some domains of the disease spectrum. A significantly higher incidence of herpes zoster was reported [90,97].

### 6.4. Therapies Contrasting JAK Activation and the Subsequent IFN Production

In view of the role played by the JAK/STAT pathway in autoimmune diseases [98], and the demonstration that filgotinib (a JAK1 inhibitor) suppressed the transcription of ISGs in human salivary gland epithelial cells [99], a growing interest has arisen in the use of kinase inhibitors in pSS. A study (NCT04496960) on the safety and tolerability of tofacinib is in progress in patients with pSS. A secondary end point of this study is to investigate the effects of tofacitinib (a JAK3-inhibitor) on salivary gland pathological changes and systemic inflammation.

A randomized, phase II, double-blind, placebo-controlled study (NCT03100942) to assess the safety and efficacy of filgotinib, together with lanraplenib (a spleen tyrosin kinase inhibitor) and tirabrutinib (a Bruton kinase inhibitor), in adults with active pSS is also ongoing.

## 7. Conclusions

Although the key role of IFN signaling in the pathogenesis of pSS has been largely proven, the precise pathological mechanisms triggered by different IFNs are far from being completely elucidated, and certainly need further investigation. The development of therapies aimed at modulating the IFN signaling in this disorder is in progress, and the preliminary results so far obtained are not conclusive and sometimes contradictory. However, all these potential new therapeutic approaches may be at risk of failure when disregarding to consider that the expression of IFNs and IFN-related genes is non-uniform in patients with different disease phenotypes. Thus, the biological processes underlying each of the disease phenotypes, including those induced by the expression of different IFNs, need to be better characterized in order to increase the probability of success of the trials assessing the efficacy of any new therapy in pSS.

## Figures and Tables

**Table 1:** Fold change values of gene expression in patients with primary Sjögren Syndrome (pSS) plus extraglandular manifestations (EGMs)- pSS-EGMs, in patients with disease limited to glandular involvement (GI) plus widespread pain (WP)-pSS-WP, and in healthy controls (HCs).

Gene	MX1	IFIT1	IFT3	IFI44	IDO1	GBP1	MIG	IP-10	P2RY14
pSS-EGMs	85.9	38.5	24.4	40.4	25.1	8.3	4.5	1.5	5.5
pSS-WP	4.2	1.7	2.0	4.8	4.1	1.2	0.6	0.3	1.3
HC	2.1	1.6	1.1	1.5	1.4	1.3	1.5	1.3	1.4

Legend. *IFN type I-induced genes:* MX1, IFN-induced GTP binding protein 1; IFIT1, IFN-induced protein with tetratricopeptide repeats 1; IFIT3, IFN-induced protein with tetratricopeptide repeats 3; IFI44, IFN-induced protein 44. *IFN type II-induced genes:* IDO1, indolamine 2,3-deoxygenase 1; GBP1, guanylate binding protein 1; MIG, C-X-C chemokine 9 (CXCL9); IP-10, C-X-C chemokine 10 (CXCL10); P2RY14, purinergic receptor 14.

**Table 2:** Targeted therapies interfering with the activation of Interferon (IFN) pathways.

Agent Code/Name	Biological Property	Mechanism of Action	Trial/Study	Status	Results	Reference
RSLV132	IgG1 fused with RNAse	degradation of circulating RNA	phase II in pSS	completed	reduction of fatigue	[84]
MEDI7734	mAb stimulating ILT7	dowregulation of TLR7 and TLR9	phase I in different autoimmune diseases	completed	no results available	clin.trials.gov: NCT02780674
BIIB059	mAb against BDCA2 antigen on pDCs	internalization of BDCA2 and inhibition of type I IFN release	phase I in SLE	completed	reduction of SLE-related skin lesions and IFN expression	[85]
BX795	Inhibition of TBK1	downregulation of IRF3 and IFR7	in vitro study on PBMCs from different autoimmune diseases	completed	reduced expression of ISGs	[86]
rontalizumab	mAb against IFN	inactivaction of IFN	phase II in SLE	completed	reduction of DA and clinical flares	[87]
sifalimumab	mAb against IFN	inactivaction of IFN	phase II in SLE	completed	reduction of DA	[88]
AMG811	mAb against IFN	inactivaction of IFN	phase Ib in SLE	completed	acceptable safety no clinical effects	[89]
anifrolumab	mAb against IFNAR	inhibition of IFN receptor	3 RCTs in SLE	completed	efficacy only in some disease manifestations	[90]
tofacitinib	JAK 3 inhibitor	suppression of ISGs transcription	phase Ib-IIa in pSS	ongoing	no results available	clin.trials.gov: NCT04496960
filgotinib	JAK 1 inhibitor	suppression of ISGs transcription	phase II in active pSS	ongoing	no results available	clin.trials.gov: NCT03100942

Legend. IgG1, Immunoglobulin G 1; pSS, primary Sjögren Syndrome; monoclobal antibody, mAb; ILT7, immunoglobulin-like transcript 7; TLR7, Toll-like receptor 7; TLR9, Toll-like receptor 9; BDCA2, Ligation of blood DC antigen 2; pDCs, plasmacytoid dendritic cells; SLE, Systemic lupus erythematosus; TBK1, TRAF family member associated NFκB activator (TANK)-binding kinase; IRF3, Interferon regulatory factor 3; IRF7, Interferon regulatory factor 7; PBMCs, peripheral blood mononuclear cells; ISGs, IFN-stimulated genes; DA, disease activity; IFNAR, Targeting IFN receptor; RCTs, randomized control trials; JAK, Janus Kinase; ISGs, IFN-stimulated genes.

## Data Availability

The datasets used and/or analyzed and reported in this review are available from the corresponding author on reasonable request.
